# Cetyltrimethylammonium Bromide (CTAB)-Loaded SiO_2_–Ag Mesoporous Nanocomposite as an Efficient Antibacterial Agent

**DOI:** 10.3390/nano11020477

**Published:** 2021-02-13

**Authors:** Aiganym Abduraimova, Anara Molkenova, Assem Duisembekova, Tomiris Mulikova, Damira Kanayeva, Timur Sh. Atabaev

**Affiliations:** 1 Department of Biology, Nazarbayev University, Nur-Sultan 010000, Kazakhstan; aiganym.abduraimova@nu.edu.kz (A.A.); aduisembekova@nu.edu.kz (A.D.); 2 Department of Chemistry, Nazarbayev University, Nur-Sultan 010000, Kazakhstan; tomiris.mulikova@nu.edu.kz

**Keywords:** mesoporous silica, silver, nanocomposite, cetyltrimethylammonium bromide, antibacterial activity

## Abstract

To date, Ag-based nanomaterials have demonstrated a high potential to overcome antibiotic resistance issues. However, bare Ag nanomaterials are prone to agglomeration in the biological environment, which results in a loss of antibacterial activity over time. Furthermore, it is still challenging to collect small-sized Ag nanomaterials right after the synthesis process. In this study, spherical-shaped Ag nanoparticles (NPs) (~6–10 nm) were attached on the surface of cetyltrimethylammonium bromide (CTAB)-loaded mesoporous silica nanoparticles (MSNs) (~100–110 nm). Antibacterial activity tests suggested that the obtained nanocomposite can be used as a highly efficient antibacterial agent against both Gram-negative and Gram-positive bacterial strains. The minimum inhibitory concentration (MIC) recalculated to pure Ag weight in nanocomposite was found to be ~1.84 µg/mL (for *Escherichia coli*) and ~0.92 µg/mL (for *Staphylococcus aureus*)—significantly smaller compared to values reported to date. The improved antibacterial activity of the prepared nanocomposite can be attributed to the even distribution of non-aggregated Ag NPs per volume unit and the presence of CTAB in the nanocomposite pores.

## 1. Introduction

According to the World Health Organization, antimicrobial resistance is an alarming issue threatening global public health [1]. From this point of view, the development of efficient antibacterial agents is considered a pivotal task for the sustainable growth of the population. Silver nanoparticles (Ag NPs) are considered to be efficient antibacterial agents with prolonged activity against various pathogens [2]. Recent studies have demonstrated that Ag NPs exhibit size- and shape-dependent antimicrobial activity [3,4,5]. In summary, small-sized Ag NPs (up to ~20 nm) demonstrated the highest bactericidal effects compared to larger counterparts. In addition, it was shown that the antibacterial activity of silver with different shapes decreases in the following sequence: spherical Ag NPs > disk Ag NPs > triangular Ag NPs [4]. Therefore, it can be concluded that spherical-shaped Ag NPs with sizes below 20 nm will demonstrate high antibacterial activity. Due to the rapid development of various synthetic procedures, the size and shape of Ag NPs can be easily altered. In particular, the synthesis of Ag NPs via chemical and physical methods was summarized in a recent review study [6]. However, the collection of small-sized Ag NPs remains a challenging problem since these tiny particles can be easily lost during a collection process.

The deposition of silver NPs on bigger-sized support particles can be considered as an effective and low-cost solution. These Ag-decorated support particles can be easily separated from the reaction media using the centrifugation process. Furthermore, one can avoid the aggregation of Ag NPs that are deposited on the surface of support particles; hence, a high amount of Ag^+^ ions can be released per volume unit. To date, low-cost and stable silica (SiO_2_) particles are often used as support or template in different applications. The simplicity of fabrication and surface modification makes them attractive for the deposition of noble metal nanoparticles. For example, Y. Tian and coworkers deposited Ag NPs (2–10 nm) on the surface of mesoporous silica particles with a size of 150–300 nm [7]. It was shown that such a structure can completely inhibit the growth of *Escherichia coli* (*E. coli*) and *Staphylococcus aureus* (*S. aureus*) strains compared to bare Ag NPs or AgNO_3_ taken with the same concentrations. J. Cui and colleagues used rice-husk-derived SiO_2_ particles (average diameter ~400 nm) for the deposition of ~10-nm-sized Ag NPs [8]. The prepared composite was found to be effective against the common rice pathogen *Xanthomonas oryzae* pv. *oryzae*. In another report, researchers successfully utilized porous SiO_2_ (150–250 nm) with Ag NPs (2–5 nm) against an *E. coli* strain [9]. It was shown that such a porous composite facilitates prolonged Ag^+^ release and improved antibacterial activity. It appears that relatively large SiO_2_ support particles have been commonly used in research. Alternatively, it will be interesting to investigate the antibacterial activity of nanocomposites smaller in size. Typically, small-sized SiO_2_ NPs can potentially host a high quantity of Ag NPs per volume unit, which in turn can boost the antimicrobial activity.

Cetyltrimethylammonium bromide (CTAB) is a cationic surfactant commonly used for the synthesis of mesoporous silica nanoparticles (MSNs) [10]. In addition, it was shown that CTAB can act as an antibacterial agent that induces superoxide stress in bacteria [11]. Thus, the main goal of this study was to investigate the synergetic antimicrobial activity of ~100–110-nm-sized CTAB-loaded MSN/Ag nanocomposite against *E. coli* and *S. aureus* bacterial strains. We showed that the minimum inhibitory concentration (MIC) recalculated to pure Ag concentration in MSN/Ag nanocomposite was lower compared to some reported values available in the literature. This feature is especially beneficial in terms of the fabrication simplicity and cost. It was concluded that a small-sized CTAB-loaded MSN/Ag nanocomposite can be utilized as a highly efficient broad-spectrum antibacterial agent.

## 2. Materials and Methods

### 2.1. Synthesis of MSNs

High purity chemicals and consumables were purchased from Merck KGaA (Darmstadt, Germany) and used as received. A hydrothermal method was utilized for the synthesis of MSNs. In brief, 60 mg of CTAB powder was dissolved in 30 mL of deionized (DI) water. The solution was heated to 75 °C, and 300 µL of NaOH solution (2M) was added under vigorous magnetic stirring. After 5 min of mixing, 500 µL of tetraethyl orthosilicate (TEOS) was added. The mixture was kept under vigorous magnetic stirring at a temperature of 75 °C for 2 h. Formed MSNs were collected by centrifugation, gently washed with DI water, and dried.

### 2.2. Synthesis of MSN/Ag

For the synthesis of MSN/Ag nanocomposite, 50 mg of MSNs was ultrasonically dispersed for several minutes in a mixture of 40 mL of DI water, 1 mL of ethanol, and 20 µL of (3-aminopropyl)triethoxysilane (APTES). A sealed glass flask with a colloidal solution was left under vigorous magnetic stirring for 30 min. Five milligrams of AgNO_3_ salt was later added, and the colloidal solution was left for 30 min under constant stirring. Finally, 100 µL of freshly prepared NaBH_4_ solution (1 mg/mL) was injected, and the final solution was stirred for 5 min. The formation of MSN/Ag nanocomposite could be observed immediately because the color of the solution turned to dark brown after the NaBH_4_ was added. The prepared nanocomposite was collected by centrifugation, gently washed with DI water, and dried.

### 2.3. Characterization

A transmission electron microscope (TEM, JEM2010F, JEOL Ltd., Tokyo, Japan) was used to observe the morphology of prepared samples. An AZtec Automated 3D energy dispersive spectroscopy (EDX) system with X-Max 150 N detector (Oxford Instruments, High Wycombe, UK) was used for the elemental analysis. Rigaku SmartLab X-ray diffractometer (XRD, Rigaku Corp., Tokyo, Japan) was employed for structural analysis. The presence of CTAB in MSN/Ag nanocomposite was confirmed by a Fourier transform infrared absorption spectrophotometer FTIR Nicolet iS5 (Thermo Fisher Scientific Inc., Waltham, MA, USA). Thermal gravimetric analysis (TGA) measurements were performed using simultaneous thermal analyzer STA-6000 (PerkinElmer Inc., Waltham, MA, USA). Samples were heated from room temperature up to 500 °C with a 10 °C min^−1^ rate. Nitrogen was used as a purge gas (flow rate 20 mL/min). Zeta-potential values of prepared samples were analyzed using Nanotrac Wave II Q (Microtrac MRB, Haan, Nordrhein-Westfalen, Germany). All measurements were carried out at a room temperature of 22 ± 1 °C.

### 2.4. Antibacterial Activity

Antibacterial activity was evaluated according to the reported protocol with some modifications [12]. Disc diffusion, minimum inhibitory concentration, and bacterial growth inhibition assay techniques were employed to investigate the antibacterial potential of MSN/Ag against *E. coli* (*Escherichia coli* (Migula) Castellani and Chalmers (ATCC 23735)) and *S. aureus* (*Staphylococcus aureus* subsp. *aureus* Rosenbach (ATCC 25923)).

### 2.5. Bacterial Inhibition Assay in Culture

In this method, bacteria were cultured in the nutrient broth (NB) medium at 37 °C for 16 h with shaking at 190 rpm. The prepared bacteria cultures were suspended in the NB and adjusted to an optical density (OD) at 600 nm of 0.1 ± 0.01 corresponding to 1 × 10^8^ CFU/mL. Next, 100 μL of the bacterial suspension was mixed with 1 mL of 1 mg/mL solution of MSN/Ag, MSNs, or penicillin in a sterilized culture tube. The bacterial suspension without the addition of any antibacterial agent was defined as the control. The tubes were incubated at 37 °C for 16 h in an orbital shaker (MaxQ 4000, Thermo Scientific, Waltham, MA, USA) at 190 rpm. The concentration of the bacterial culture was monitored every 3 h by measuring OD_600_ on a spectrophotometer (Jenway 632621, Fisher Scientific, Waltham, MA, USA).

### 2.6. Assessment of Bacterial Viability

Bacterial viability was assessed by visual inspection of the colony-forming units in the presence of antibacterial agents. After 12 h incubation, 10-fold serial dilutions were prepared, and 100 μL of the diluted bacterial suspension (10^−7^) was plated onto the Trypticase soy agar (TSA). All plates were left for 16 h incubation at 37 °C. Results of the viable cell count were directly shown in the form of agar plate photographs obtained by UVP Colony Doc-IT (Analytik Jena, Jena, Germany).

### 2.7. Disc Diffusion Method

Mueller–Hinton agar (MHA) plates were cotton-swabbed with the bacterial suspension at 0.1 OD_600_. Sterile filter discs (d = 130 mm, Whatman, UK) were placed onto MHA plates. Next, 10 μL of MSN/Ag or MSNs at different concentrations (0, 0.25, 0.5, 1, 2 mg/mL) were added on top of the discs. Plates were incubated at 37 °C for 16 h. The inhibition zone diameter was measured using UVP Colony Doc-IT (Analytik Jena, Jena, Germany).

### 2.8. Minimum Inhibitory Concentration (MIC)

A sterile 96-well plate was used to determine the MICs of antibacterial agents. Serial 2-fold dilutions were performed using 2 mg/mL of MSN/Ag solution in brain heart infusion broth to generate the concentrations in the range from 0 to 1.0 mg/mL. Next, 10 μL of a bacterial suspension at 0.1 OD_600_ was added into wells containing antibacterial agents. Then, plates were incubated at 37 °C for 18 h with a constant agitation rate of 150 rpm. After the incubation, the MICs were estimated by spectrophotometric analysis and visual inspection. The plates were read at OD_600_ using a Varioskan microplate reader (Varioskan Flash, Thermo Fisher Scientific, Vantaa, Finland). The MIC was defined as the lowest concentration of MSN/Ag at which the bacterial growth was effectively suppressed.

### 2.9. Statistical Analysis

All experiments were repeated three times to ensure the reproducibility of the results. Results are expressed as the mean ± SD. *p* values were determined using two-way ANOVA analysis (* *p* < 0.05).

## 3. Results

**Figure 1:**
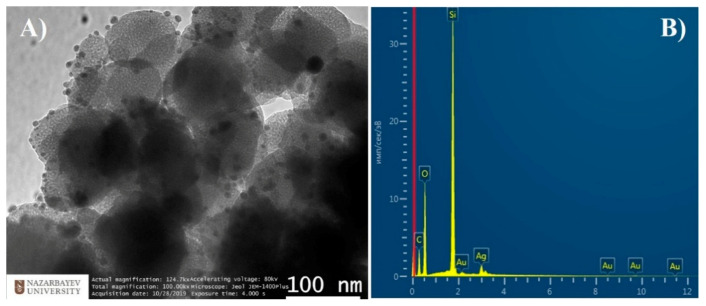
(**A**) TEM image and (**B**) EDX analysis of mesoporous silica nanoparticle (MSN)/Ag nanocomposite.

TEM analysis was used for morphological analysis of prepared CTAB-loaded MSN/Ag nanocomposite. Figure 1A shows that MSNs appeared as light gray quasispherical particles (~100–110 nm) with ordered mesoporous structure. Ag NPs (mainly ~6–10 nm) appeared as dark gray nanoparticles because of the density difference between SiO_2_ and Ag. One can easily observe Ag NPs evenly distributed on the MSN surface, suggesting the formation of the MSN/Ag nanocomposite. EDX analysis (Figure 1B) confirmed the presence of all constituting elements, i.e., Si, O, C, and Ag, in the MSN/Ag nanocomposite. As a strong reducing agent, NaBH_4_ immediately reacts with AgNO_3_ to form Ag NPs that are fully deposited on the MSNs (i.e., around 63.5 µg of Ag per 1 mg of MSN) thanks to surface silanization with APTES. A weak Au signal appeared due to a thin gold coating created during the sample preparation process for EDX analysis.

**Figure 2:**
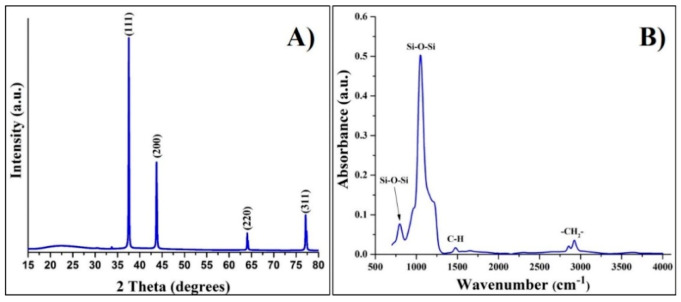
(**A**) XRD and (**B**) FTIR analysis of MSN/Ag nanocomposite.

XRD analysis was further conducted to confirm the formation of pure Ag NPs on the surface of MSNs. Figure 2A shows a typical XRD pattern of the MSN/Ag nanocomposite. Four strong peaks were detected, corresponding to the (111), (200), (220), and (311) crystal planes of face-centered cubic (fcc) Ag structure (JCPDS: 89–3722) [13]. A broad and low-intensity peak observed between ~15–30 2-theta degrees is associated with amorphous MSNs. No other peaks were detected, highlighting the deposition of pure Ag NPs on the MSN surface. The presence of CTAB in the MSN/Ag nanocomposite pores was further confirmed by FTIR analysis. Two characteristic absorption bands located at 798 and 1075 cm^−1^ can be attributed to symmetric and asymmetric Si–O–Si vibrations [14]. The band located in the region of 1431–1507 cm^−1^ appeared due to CTAB asymmetric and symmetric C–H scissoring of H_3_C–N^+^ [15]. Another two absorption bands located at 2849 and 2917 cm^−1^ were attributed to asymmetric and symmetric –CH_2_– stretching of the CTAB chain, respectively [15].

TGA analysis was further performed to quantify the CTAB amount in bare MSNs and MSN/Ag nanocomposite. Figure S1 (Supplementary Materials) shows typical TGA curves measured for bare MSNs and MSN/Ag. In both cases, no weight drop was observed up to 120 °C, suggesting that the samples had no adsorbed moisture on their surface. These results are also in good agreement with the FTIR data. The obvious weight loss for both samples was observed from 150 to 350 °C because of CTAB removal. The nanocomposite was also analyzed by FTIR after the calcination at 500 °C (Figure S2, Supplementary Materials). It is evident that all CTAB-related peaks disappeared, suggesting a complete CTAB removal. The calculated weight loss was found to be 24.97% and 22.19% for bare MSNs and MSN/Ag, respectively. It was found that MSN/Ag nanocomposite had a lower CTAB amount compared to MSNs. The plausible explanation is that some CTAB was lost during the Ag NP attachment procedure. Zeta-potential measurements of bare MSNs (+37.3 mV) and MSN/Ag (+31.1 mV) also showed a slight charge difference in prepared samples. Nevertheless, MSN/Ag nanocomposite still can bind to negatively charged bacterial cells via electrostatic interaction. The mesoporous structure of MSN/Ag was further confirmed by nitrogen adsorption–desorption experiments. Figure S3 (Supplementary Materials) shows a typical nitrogen adsorption–desorption isotherm for MSN/Ag taken after the synthesis process. The MSN/Ag had a Brunauer–Emmet–Teller (BET) specific surface area of ~49.71 ± 0.83 m^2^/g. However, the BET specific surface area of the same MSN/Ag after firing at 500 °C for 1 h increased to ~651.08 ± 1.66 m^2^/g (Figure S4, Supplementary Materials). The obvious increase of specific surface area suggested that CTAB was localized in the pores of MSN/Ag.

**Figure 3:**
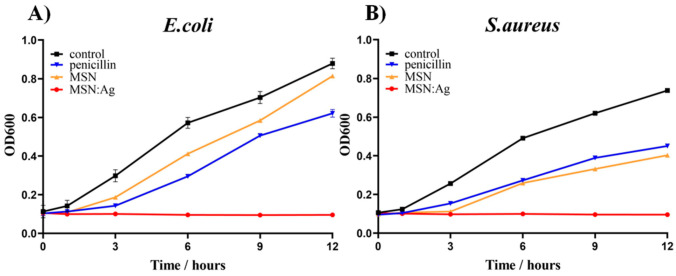
Inhibitory effects of MSN/Ag, MSNs, and penicillin at 1 mg/mL on the growth of *E. coli* (**A**) and *S. aureus* (**B**) in nutrient broth (NB) liquid media. Bacterial growth was monitored by measuring OD_600_. Each condition was prepared in triplicate. Each data point represents an average of three parallel samples, and the error bars indicate 1 standard deviation from the mean.

The antibacterial activity of MSN/Ag was evaluated against Gram-negative *E. coli* and Gram-positive *S. aureus* bacteria. For comparison purposes, the antibacterial effects of penicillin taken at the same concentration and bare MSNs were studied as well. The benefit of the mesoporous structure in MSN/Ag NPs is associated with CTAB storage that facilitates closer bacterial contact with nanocomposite because of the electrostatic interaction. In addition, the release of CTAB from pores can also enhance the antibacterial activity of the nanocomposite. Ag NPs have broad-spectrum inhibitory and bactericidal activity by altering the membrane permeability respiration of *E. coli* and inhibiting the DNA replication of *S. aureus* [16]. It should be noted that the antibacterial properties of Ag NPs are associated with the release of Ag^+^ ions, which may be disruptive to the cell walls of various strains. In particular, Ag^+^ ions can be attached to the negatively charged protein and nucleic acid, which causes structural changes in the cell wall and membrane. Ag NPs damage cellular membranes and induce the formation of reactive oxygen species (ROS), which leads to ribosome destabilization and DNA damage [17]. Figure 3 shows the inhibitory effects of MSN/Ag, MSNs, and penicillin on the bacteria growth, which was monitored every 3 h over a 12 h period. The bacterial growth was assessed by an OD_600_ measurement that was conducted in triplicate, and only negligible deviation was observed. It is clear that penicillin and MSNs exhibit moderate inhibition over 12 h incubation. In comparison to penicillin, MSNs demonstrated a lower inhibition rate against *E. coli* (OD_600_ = 0.814) than *S. aureus* (OD_600_ = 0.403). However, MSN/Ag was capable of maintaining the continuous inhibition of the bacterial growth for both bacterial strains, and OD_600_ remained stable at around 0.1 value. The high antibacterial activity of MSN/Ag could be attributed to the synergetic effects of CTAB and Ag presence in the nanocomposite. Figure S5 (Supplementary Materials) shows typical SEM images of *E. coli* and *S. aureus* before and after contact with MSN/Ag. It is clear that the shape of the cells was dramatically changed, suggesting their effective interaction with MSN/Ag. The *S. aureus* cells were affected more strongly (lysis can be observed) when compared to *E. coli* cells, corresponding well with the data presented in Figure 3.

**Figure 4:**
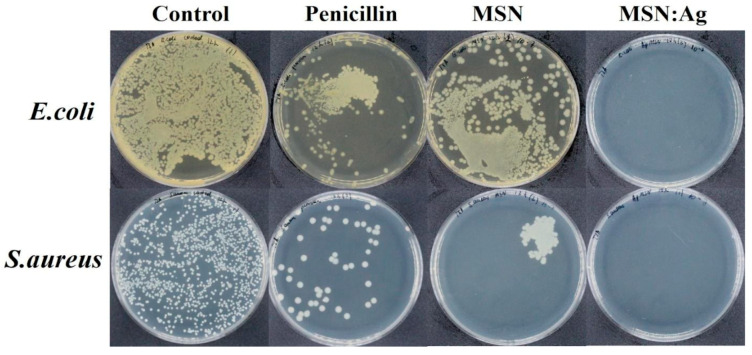
Bacterial viability assay using control, penicillin, MSNs, and MSN/Ag at 1 mg/mL after 12 h incubation with 1 × 10^8^ CFU of *E. coli* and *S. aureus*.

Figure 4 shows bacterial inhibition photographs obtained after 12 h incubation, which agree with the OD_600_ measurements. The MSN/Ag displayed a complete inhibition of bacterial growth as no colonies were observed after 12 h incubation. In the case of penicillin and MSNs, several bacterial colonies were formed, proving their moderate bactericidal properties over prolonged incubation in liquid media.

**Figure 5:**
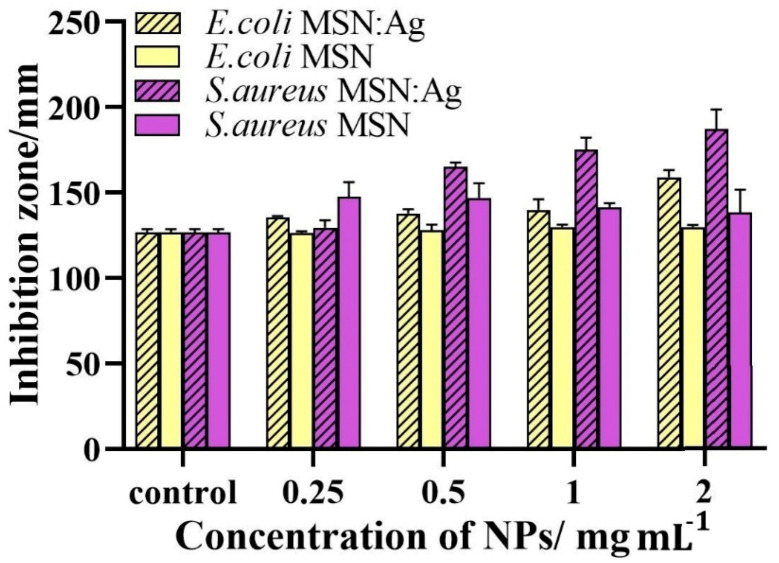
The zones of inhibition generated by MSN/Ag and MSNs against *E. coli* and *S. aureus* tested at different concentrations after 16 h incubation. Each data point represents an average of three parallel samples, and the error bars indicate 1 standard deviation from the mean.

The disc diffusion method was further employed to study the susceptibility of bacteria to MSNs and MSN/Ag. It should be noted that the disc diffusion method is known as a qualitative method since this method has numerous limitations and is mainly used to verify the presence or absence of the antimicrobial agent. Figure 5 shows that MSN/Ag exhibits better antibacterial properties against *S. aureus* as compared to *E. coli*. The zones of inhibition of MSN/Ag (2 mg/mL) against *E. coli* and *S. aureus* were determined as 159.1 and 187.2 mm, respectively. These results suggest that Gram-negative *E. coli* is less susceptible to MSN/Ag NPs compared to Gram-positive *S. aureus*. One can see that no significant changes in MSN inhibition zones against *E. coli* were observed over a wide range of concentrations, indicating their limited activity against this pathogen. In contrast, *S. aureus* was susceptible to both MSNs and MSN/Ag. Such discrepancy could be attributed to the different membrane structures of these two strains of bacteria [12]. Our findings are in agreement with another report [18] demonstrating that the SiO_2_–Ag-based structure exhibits better antibacterial activity against Gram-positive bacteria compared to Gram-negative bacteria. This is because the outer cell membrane of Gram-negative bacteria is predominantly composed of lipopolysaccharides, lipoproteins, and phospholipids that can trap positively charged ions/molecules and prevent their easy penetration [9], whereas the outer cell membrane of Gram-positive bacteria is built from a porous layer consisting of peptidoglycan and teichoic acid. These pores are large enough to allow an unobstructed penetration of molecules and nanoparticles that cause bacterial death [19].

Finally, the MIC values were evaluated and recalculated to the weight of pure Ag. The experiments showed that the MICs of MSN/Ag were 31.25 µg (~1.84 µg of Ag) and 15.63 µg (~0.92 µg of Ag) for *E. coli* and *S. aureus*, respectively. Table 1 summarizes the MIC values for some bare Ag NPs and SiO_2_–Ag structures reported in literature recently. The MIC values for MSN/Ag were sufficiently smaller compared to any available data in the literature values so far. It can be concluded that the synergetic action of CTAB and Ag results in improved antibacterial properties of the nanocomposite. Furthermore, the proposed method can simplify the overall fabrication process, as there is no need to remove CTAB from MSN pores. Moreover, the proposed method can be beneficial in terms of fabrication cost since a lower amount of Ag precursor is required for the synthesis. 

## 4. Conclusions

In summary, a CTAB-loaded MSN/Ag nanocomposite was successfully prepared for potential antibacterial applications. Antibacterial activity results and MIC comparison with available literature results indicate the efficient antibacterial activity of the prepared MSN/Ag nanocomposite. The improved antimicrobial activity of MSN/Ag could be attributed to several factors, such as the presence of CTAB in composite, electrostatic interaction, and even distribution of small Ag NPs (~6–10 nm) on the surface of MSNs. It was concluded that MSN/Ag demonstrates great potential as a candidate for the development of products aiming to prevent the spread of drug-resistant pathogens.

## Figures and Tables

**Table 1:** Minimum inhibitory concentration (MIC) data for some Ag and SiO_2_–Ag structures.

Composition	MIC Recalculated to Pure Ag	Reference
mSiO_2_@NH_2_@Ag (~140 nm)	*E. coli* (10.12 µg/mL)	[18]
Ag NPs (5–20 nm)	*E. coli* (64 µg/mL), *S. aureus* (256 µg/mL)	[20]
Bio-Ag NPs, (8–48 nm)	*S. aureus* (~6.25 µg/mL)	[21]
Ag NPs@WMSs (~150 nm)	*E. coli* (~3 µg/mL), *S. aureus* (~4 µg/mL)	[22]
CTAB-MSN/Ag (~120 nm)	*E. coli* (~2 µg/mL), *S. aureus* (~1 µg/mL)	This study

## Data Availability

The data presented in this study are available on a reasonable request from the corresponding authors.

## Supplementary Materials

The following are available online at https://www.mdpi.com/2079-4991/11/2/477/s1, Figure S1: TGA analysis, Figure S2: FTIR analysis, Figure S3: Nitrogen adsorption–desorption analysis #1, Figure S4: Nitrogen adsorption–desorption analysis #2, Figure S5: SEM images of bacteria before and after contact with MSN/Ag.
