# Supplementary material for: Cetyltrimethylammonium Bromide (CTAB)-Loaded SiO2–Ag Mesoporous Nanocomposite as an Efficient Antibacterial Agent
Source: Nanomaterials (Basel). 2021 Feb 13;11(2):477. doi: 10.3390/nano11020477 (PMC7917824; doi:10.3390/nano11020477)
Supplement: Supplementary file 1 [file nanomaterials-11-00477-s001.pdf]

Supporting Information

Cetyltrimethylammonium Bromide (CTAB)-Loaded SiO_2–Ag Mesoporous Nanocomposite as an Efficient Antibacterial Agent

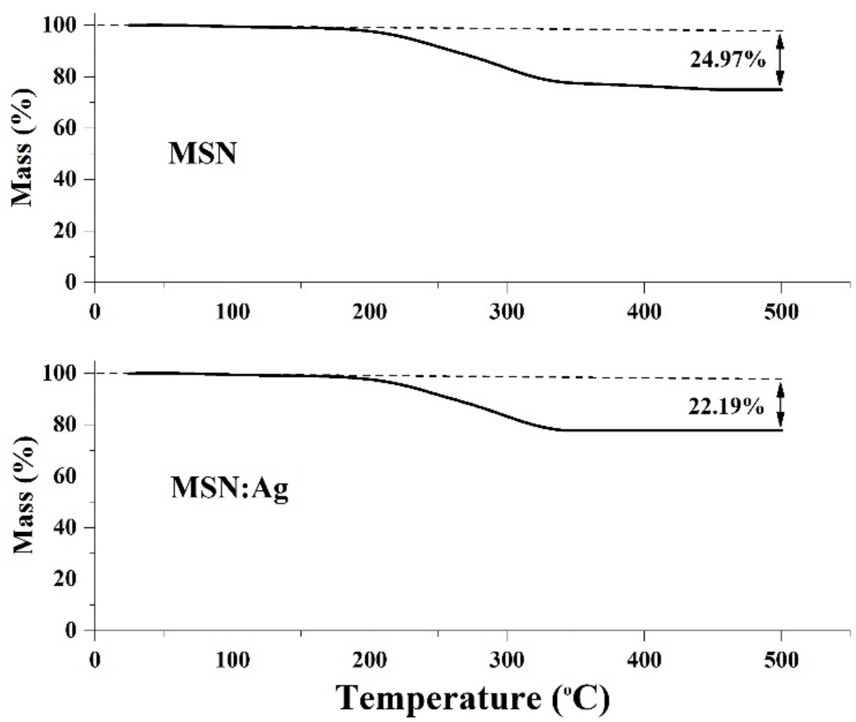

Figure S1. TGA curves for bare MSN and MSN:Ag.

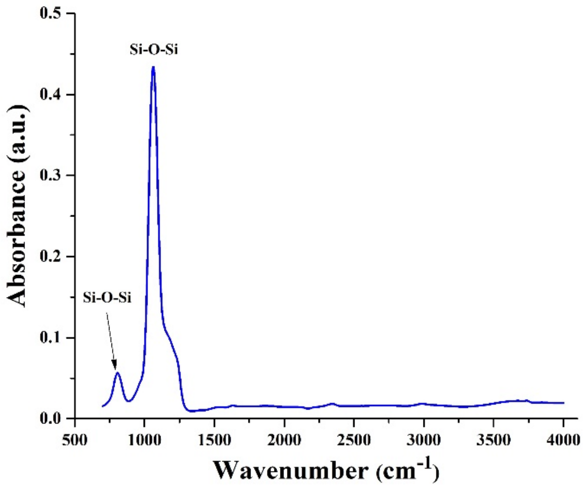

Figure S2. FTIR analysis of MSN:Ag nanocomposite after the TGA.

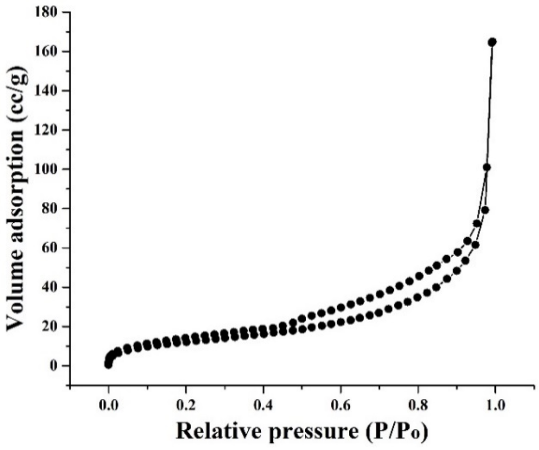

Figure S3. Nitrogen adsorption-desorption isotherm of MSN:Ag after the synthesis process.

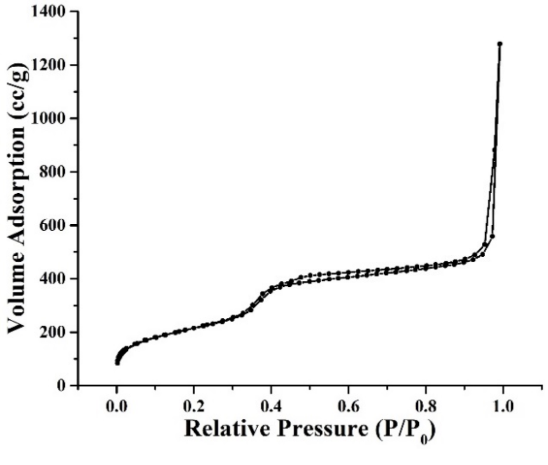

Figure S4. Nitrogen adsorption-desorption isotherm of MSN:Ag after the calcination at 500 ^{o}C.

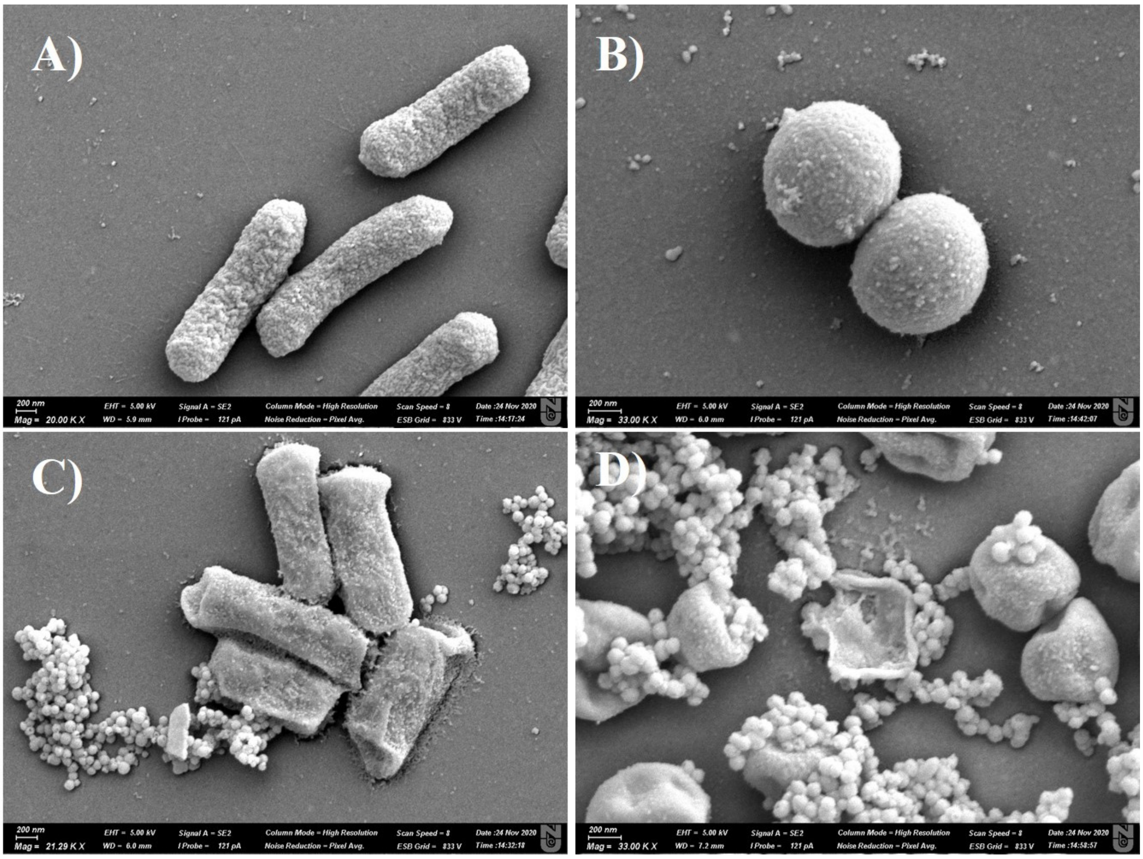

Figure S5. SEM images of control *E.coli* (A) and *S.aureus* (B). SEM images of MSN:Ag incubated with *E.coli* (C) and *S.aureus* (D).
